# Glesatinib, a c-MET/SMO Dual Inhibitor, Antagonizes P-glycoprotein Mediated Multidrug Resistance in Cancer Cells

**DOI:** 10.3389/fonc.2019.00313

**Published:** 2019-04-25

**Authors:** Qingbin Cui, Chao-Yun Cai, Hai-Ling Gao, Liang Ren, Ning Ji, Pranav Gupta, Yuqi Yang, Suneet Shukla, Suresh V. Ambudkar, Dong-Hua Yang, Zhe-Sheng Chen

**Affiliations:** ^1^School of Public Health, Guangzhou Medical University, Guangdong, China; ^2^Department of Pharmaceutical Sciences, College of Pharmacy and Health Sciences, St. John's University, Queens, NY, United States; ^3^Department of Histology and Embryology, Clinical Medical College, Weifang Medical University, Weifang, China; ^4^Tianjin Key Laboratory on Technologies Enabling Development of Clinical Therapeutics and Diagnostics, School of Pharmacy, Tianjin Medical University, Tianjin, China; ^5^Laboratory of Cell Biology, Center for Cancer Research, National Cancer Institute, NIH, Bethesda, MD, United States

**Keywords:** multidrug resistance, P-gp, glesatinib, reversal effects, mechanism

## Abstract

Multidrug resistance (MDR) is one of the leading causes of treatment failure in cancer chemotherapy. One major mechanism of MDR is the overexpressing of ABC transporters, whose inhibitors hold promising potential in antagonizing MDR. Glesatinib is a dual inhibitor of c-Met and SMO that is under phase II clinical trial for non-small cell lung cancer. In this work, we report the reversal effects of glesatinib to P-glycoprotein (P-gp) mediated MDR. Glesatinib can sensitize paclitaxel, doxorubicin, colchicine resistance to P-gp overexpressing KB-C2, SW620/Ad300, and P-gp transfected Hek293/ABCB1 cells, while has no effect to their corresponding parental cells and negative control drug cisplatin. Glesatinib suppressed the efflux function of P-gp to [^3^H]-paclitaxel and it didn't impact both the expression and cellular localization of P-gp based on Western blot and immunofluorescent analysis. Furthermore, glesatinib can stimulate ATPase in a dose-dependent manner. The docking study indicated that glesatinib interacted with human P-gp through several hydrogen bonds. Taken together, c-Met/SMO inhibitor glesatinib can antagonize P-gp mediated MDR by inhibiting its cell membrane transporting functions, suggesting new application in clinical trials.

## Introduction

Multidrug resistance (MDR) is the one of the major challenges in cancer treatment (1). MDR refers to a phenomenon that cancer cell once becomes resistant to one chemotherapeutic, accompanied by cross resistant to other chemotherapeutics that are structurally and mechanistically different (2). MDR is one of the major causes of failure in cancer treatment. The mechanisms of MDR involve dynamic ATP-binding cassette (ABC) transporters (3, 4), oncogenes mutations (5), microenvironment changes (6), reprogramed cancer cell metabolism (7, 8), efficient DNA repairing (9, 10), survived cancer stem cells (11, 12), and activated detoxifying systems (13, 14). Novel effective remedies are urgently needed to circumvent MDR.

**Figure 1:**
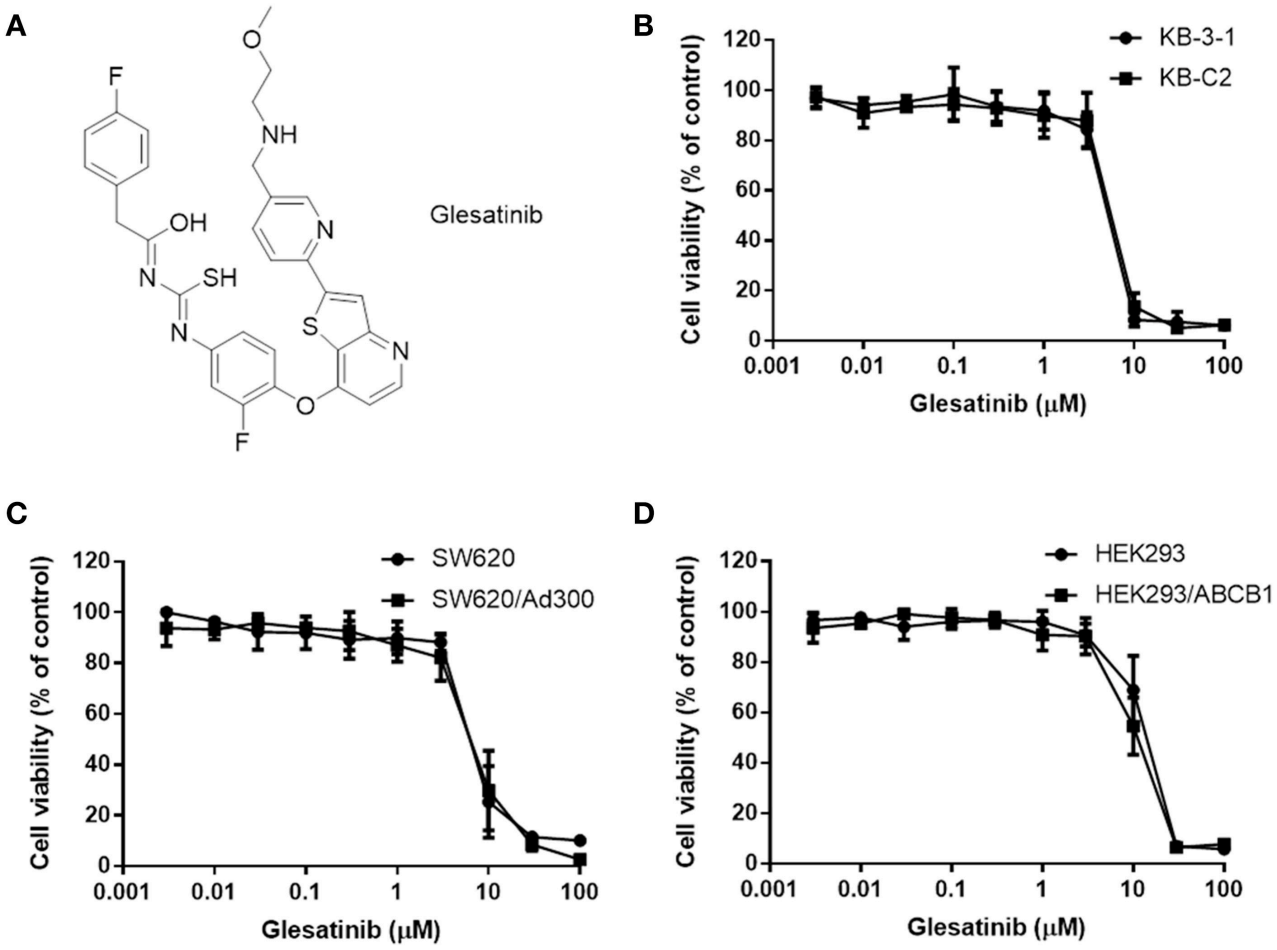
The structure of glesatinib and its cytotoxic effects to three P-gp overexpressing cancer cells. **(A)** Chemical structure of glesatinib. **(B)** Concentration-dependent viability curves for KB-3-1 and KB-C2 cell lines incubated with different concentration of glesatinib for 72 h. **(C)** Concentration-dependent viability curves for SW620 and SW620/Ad300 cell lines incubated with different concentration of glesatinib for 72 h. **(D)** Concentration-dependent viability curves for HEK293/pcDNA3.1 and HEK293/ABCB1 cells incubated with different concentration of glesatinib for 72 h. The cell viability was determined by MTT assay. Data are expressed as mean ± SD, and representative of three independent experiments in triplicate are shown.

ABC transporters are a group of active transporter proteins that have diverse functions and are present in the membrane of both prokaryotes and eukaryotes, acting as protecting enzymes against xenobiotic, including many chemotherapeutics (15, 16). One of the most well studied ABC transporters is P-glycoprotein (P-gp), which is encoded by ABCB1 genes. P-gp contributes in pumping out many different kinds of anticancer drugs, namely, taxanes, anthracyclines, vinca alkaloids, and epipodophyllotoxins (17–24). To counteract the negative regulation of chemotherapy by P-gp, three generations of inhibitors (both specific and non-specific) have been developed and some of them have been introduced into clinical trials (25). However, due to unexpected adverse effects or severely drug-drug interaction, none of them have been approved by FDA (3, 26). There is an unmet need for effective and safe reversal agents for clinical use. Recently, certain tyrosine kinase inhibitors (TKIs) have been found to exert MDR reversal effect via regulating P-gp at non-toxic concentration (27–31), suggesting new regimens in the treatment of resistant cancer. TKI glesatinib (Figure 1A), a c-MET/SMO dual inhibitor (32, 33), is now under Phase II clinical trials in combination with Nivolumab in treatment of the non-small cell lung cancer (NSCLC). More importantly, we found that glesatinib can antagonize P-gp mediated MDR. Here, we report the reversal effects of glesatinib and the underlying mechanisms.

## Materials and Methods

### Chemicals

Glesatinib (99% purity as measured by high performance liquid chromatography) was purchased from ChemieTek (Indianapolis, IN). Dulbecco's modified Eagle's Medium (DMEM), bovine serum albumin (BSA), fetal bovine serum (FBS), penicillin/streptomycin and trypsin 0.25% were purchased from Hyclone (GE Healthcare Life Science, Pittsburgh, PA). The monoclonal antibodies for ABCB1 (C219) and GAPDH (MA5-15738), Alexa Fluor 488 conjugated goat anti-mouse IgG secondary antibody were purchased from Thermo Fisher Scientific Inc (Rockford, IL), dimethylsulfoxide (DMSO), 3-(4,5-dimethylthiazol-yl)-2,5-diphenyltetrazolium bromide (MTT), Triton X-100, 4',6-diamidino-2-phenylindole (DAPI), paraformaldehyde, paclitaxel, doxorubicin, colchicine, cisplatin, verapamil and Ko 143 were purchased from Sigma-Aldrich (St. Louis, MO). [^3^H]-paclitaxel (15 Ci/mmol) was purchased from Moravek Biochemicals, Inc (Brea, CA). All other chemicals were purchased from Sigma Chemical Co (St. Louis, MO).

### Cell Lines and Cell Culture

The human epidermoid carcinoma cell line KB-3-1 and its colchicine-selected P-gp-overexpressing KB-C2 cells, the human colon cancer cell line SW620 and its doxorubicin-selected P-gp-overexpressing SW620/Ad300 cells, the NSCLC cell line NCI-H460 and its mitoxantrone-selected ABCG2-overexpressing NCI-H460/MX20 cells, were used for P-gp and ABCG2 reversal study, respectively. The HEK293/pcDNA3.1, HEK293/ABCB1 cells lines were established by transfecting HEK293 cells with either the empty pcDNA3.1 vector or the vector containing full length ABCB1 (HEK293/ABCB1), and were cultured in a medium containing 2 mg/mL of G418. All cell were cultured at 37°C, using 5% CO_2_ with DMEM containing 10% FBS and 1% penicillin/streptomycin. All drug resistant cell lines were grown as adherent monolayer in a drug-free culture media for more than 2 weeks prior to their use.

### Cytotoxicity and Reversal Experiments

The cytotoxicity and reversal experiments of glesatinib to KB-3-1, KB-C2, SW620, SW620/Ad300, HEK293/pcDNA3.1, HEK293/ABCB1 cells were performed by using the MTT colorimetric assay (34). For reversal experiments, the applied concentrations of glesatinib were 1 and 3 μM according to the results of cytotoxicity experiments. All of the experiments were repeated at least three times, and the mean and standard deviation (SD) values were calculated. Verapamil (3 μM) was used as a positive control inhibitor of P-gp, Ko 143 was used as a positive control inhibitor of ABCG2, cisplatin, a non-P-gp substrate, was used as a negative control.

### Western Blot Analysis

Dose-dependent (0, 0.3, 1, 3 μM) and time-dependent (0, 24, 48, 72 h) of glesatinib on the expression of P-gp were determined. Twenty microgram protein cell lysates were loaded in each lane. The presence of P-gp was determined using monoclonal antibody C219 (dilution 1:200). GAPDH was used to confirm equal loading in each lane in the samples prepared from cell lysates. The resulting protein bands were quantified by using Image J software. The detailed protocol of Western blot analysis was carried out as previously described (35).

### Immunofluorescence Analysis

SW620, SW620/Ad300 cells were seeded (1 × 10^4^/well) in 24-well plates and cultured at 37°C for 24 h, followed by incubation with 3 μM glesatinib for 0, 24, 48, and 72 h, respectively. Then cells were fixed in 4% paraformaldehyde for 5 min and permeabilized by 0.1% Triton X-100 for 5 min before blocked with 6% BSA for 1 h at 37°C. The presence of P-gp was determined using monoclonal antibody F4 (dilution 1:1000) for incubation at 4°C overnight. Alexa Fluor 488 conjugated secondary antibody (1:1000) was used for incubation at 37°C for 1 h. After washing with iced PBS, DAPI (1 μg/mL) was used to counterstain the nuclei. Immunofluorescence images were collected using an EVOS FL Auto fluorescence microscope (Life Technologies Corporation, Gaithersburg, MD).

### ATPase Assay

The vanadate-sensitive ATPase activity of ABCB1 in membrane vesicles of High Five insect cells was measured as previously described (36). Briefly, the membrane vesicles (10 μg of protein) were incubated in ATPase assay buffer [composed by 50 mmol/L MES (pH 6.8), 50 mmol/L KCl, 5 mmol/L sodium azide, 2 mmol/L EGTA, 2 mmol/L DTT, 1 mmol/L ouabain, and 10 mmol/L MgCl_2_] with or without 0.3 mmol/L vanadate at 37°C for 5 min, then were incubated with different concentrations (ranging from 0 to 40 μM) of glesatinib at 37°C for 3 min. The ATPase reaction was induced by the addition of 5 mM of Mg-ATP, and the total volume was 0.1 mL. After incubation at 37°C for 20 min, the reaction was allowed to continue for another 20 min at 37°C and then terminated by adding 100 μL of a 5% SDS solution to the reaction mix. The amount of inorganic phosphate (IP) release was detected at 880 nm using a spectrophotometer.

### [^3^H]-Paclitaxel Accumulation and Efflux Assay

Since glesatinib reversed MDR mediated by P-gp, the reversal mechanism may be related to change of the protein expression or location of P-gp, we used the drug accumulation and efflux assays to determine the reversal mechanism as previously described (27). The accumulation and efflux of [^3^H]-paclitaxel in KB-3-1 and KB-C2 cells were measured in the absence or presence of glesatinib (1, 3 μM), and verapamil (3 μM) was used as positive control.

### Molecular Modeling of Human ABCB1 Homology Model

To reveal more details of the interaction between glesatinib and P-gp, we conducted docking study. All docking experiments were performed following the reported protocols with software Schrodinger 2018–1 (Schrödinger, LLC, New York, NY, 2018) on a Mac Pro 6-core Intel Xenon X5 processor with Macintosh Operating System (OS X El Capitan) (28, 37). Ligand preparation was essentially performed. Human P-gp homology model (4M1M) was established by Dr. S. Aller based on improved mouse P-gp (3G5U). Single-wavelength anomalous diffraction (SAD) phasing was conducted to the full 3.8 Å resolution of the dataset. Non-crystallographic symmetry (NCS) operators were determined from the mouse P-gp structure with the phenix.python script simple_ncs_from_pdb.py. Refinement was conducted with phenix.refine using NCS and secondary structure restraints, restraining NCS-related B-factors, group B-factor and individual B-factor (38). The centroid of some important residues including H61, G64, L65, M68, L339, A342, L975 C343, F942, T945, Q946, Y950, L975, V982, and A985 (39–41). Glide XP docking was performed and the receptor grid for induced-fit docking (IFD) was generated by selecting residues. Then IFD was conducted with the default protocol.

### Statistical Analysis

All data are expressed as the mean ± SD. All experiments were repeated at least three times and the data were analyzed using a one-way or two-way ANOVA by GraphPad Prism 7.00 software. Differences were considered significant when *P* < 0.05.

## Results

### Glesatinib Antagonized MDR in P-gp Overexpressing Cancer Cells

First, the cytotoxicity of glesatinib to P-gp overexpressing cancer cells KB-C2, SW620/Ad300, HEK293/ABCB1, and their parent cells KB-3-1, SW620, HEK293 cells were determined by MTT assay. As shown in Figures 1B–D, the IC_50_s fell between 5 and 10 μM. Therefore, the non-toxic concentration (IC_20_) of glesatinib applied in the reversal effects evaluation were 1 and 3 μM.

The reversal effects of glesatinib to P-gp substrates, including doxorubicin, paclitaxel and colchicine were further tested in the aforementioned cancer cells. The non-selective P-gp inhibitor, verapamil was used as a positive control (42), and non-substrate cisplatin was used as a negative control (43). Pretreatment with or without glesatinib with these substrates to P-gp overexpressing cancer cells and their sensitive parent cells were tested to obtain their IC_50_s.

As shown in Tables 1, 2, the parent cells were sensitive to doxorubicin, paclitaxel and colchicine, and the IC_50_s were as low as nano-mole. While P-gp overexpressing cancer cell exhibited resistant properties to these chemotherapeutics, resistance fold ranged from 77 to 438. Pretreatment with glesatinib significantly lowered the IC_50_s of all these three chemotherapeutics to resistant cancer cells. More importantly, glesatinib exhibited similar re-sensitizing effects to P-gp transfected HEK293/ABCB1 cells, suggesting its mechanisms of re-sensitizing to chemotherapeutics were directly or indirectly related to P-gp. In addition, in ABCG2 overexpressing cancer cells NCI-H460/MX20 cells, gleasatinib failed to reverse topotecan (an ABCG substrate) resistance (Table 2). These results indicated that glesatinib could antagonize cancer MDR mediated by P-gp, but not MDR mediated by ABCG2.

**Table 1:** Glesatinib sensitized paclitaxel, colchicine, and doxorubicin to P-gp-overexpressing cell lines (KB-C2 and HEK293/ABCB1 cells).

**Treatment**	**IC_50_± SD^a^ (RF^b^)**			
	**KB-3-1 (μM)**	**KB-C2 (μM)**	**HEK293 (μM)**	**HEK293/ABCB1 (μM)**
Paclitaxel + Gle (1 μM) + Gle (3 μM) + Vera (3 μM)	0.004 ± 0.002 (1.00) 0.004 ± 0.001 (1.00) 0.003 ± 0.001 (0.75) 0.003 ± 0.001 (0.75)	1.755 ± 0.057 (438.75) 0.220 ± 0.026 (55)^*^ 0.015 ± 0.001 (3.75)^*^ 0.010 ± 0.002 (2.5)^*^	0.073 ± 0.027 (1.00) 0.122 ± 0.050 (1.67) 0.100 ± 0.020 (1.37) 0.068 ± 0.003 (0.95)	3.757 ± 0.312 (51.46) 0.255 ± 0.084 (3.49)^*^ 0.047 ± 0.004 (0.64)^*^ 0.094 ± 0.003 (1.9)^*^
Doxorubicin + Gle (1 μM) + Gle (3 μM) + Vera (3 μM)	0.032 ± 0.013 (1.00) 0.029 ± 0.003 (0.91) 0.028 ± 0.004 (0.88) 0.024 ± 0.006 (0.75)	2.504 ± 0.487 (78.25) 0.118 ± 0.061 (3.69)^*^ 0.023 ± 0.010 (0.72)^*^ 0.024 ± 0.005 (0.75)^*^	0.061 ± 0.020 (1.00) 0.060 ± 0.029 (0.98) 0.066 ± 0.009 (1.08) 0.061 ± 0.008 (1.00)	0.631 ± 0.150 (10.34) 0.072 ± 0.006 (1.18)^*^ 0.064 ± 0.021 (1.05)^*^ 0.084 ± 0.009 (1.38)^*^
Colchicine + Gle (1 μM) + Gle (3 μM) + Vera (3 μM)	0.009 ± 0.002 (1.00) 0.006 ± 0.002 (0.67) 0.007 ± 0.001 (0.78) 0.009 ± 0.001 (1.00)	3.231 ± 0.260 (359.00) 0.993 ± 0.183 (110.33)^*^ 0.088 ± 0.020 (9.78)^*^ 0.116 ± 0.035 (12.89)^*^	0.066 ± 0.001 (1.00) 0.058 ± 0.007 (0.88) 0.048 ± 0.009 (0.73) 0.056 ± 0.006 (0.85)	1.538 ± 0.090 (23.30) 0.126 ± 0.106 (1.91)^*^ 0.047 ± 0.021 (0.71)^*^ 0.050 ± 0.008 (0.76)^*^
Cisplatin + Gle (1 μM) + Gle (3 μM) + Vera (3 μM)	2.508 ± 0.432 (1.00) 1.990 ± 0.452 (0.79) 2.031 ± 0.364 (0.81) 2.309 ± 0.641 (0.92)	3.027 ± 0.343 (1.21) 2.676 ± 0.443 (1.07) 2.120 ± 0.152 (0.85) 2.098 ± 0.230 (0.84)	2.660 ± 0.430 (1.00) 1.982 ± 0.253 (0.75) 1.903 ± 0.361 (0.72) 2.388 ± 0.452 (0.90)	3.336 ± 0.451 (1.25) 3.272 ± 0.254 (1.23) 3.394 ± 0.353 (1.28) 3.115 ± 0.433 (1.17)

* *P < 0.05 vs. no inhibitor group*.

a *IC_50_ values represented the mean ± SD of three independent experiments performed in triplicate*.

b *Resistance fold (RF) was calculated by dividing the IC50 values of substrates in the presence or absence of an inhibitor by the IC_50_ values of parental cells without an inhibitor. Gle, Glesatinib; Vera, verapamil*.

**Table 2:** Glesatinib sensitized paclitaxel, colchicine, and doxorubicin to P-gp-overexpressing cell line (SW620/Ad300 cells), but not topotecan to ABCG2-overexpressing cells (NCI-H460/MX20 cells).

**Treatment**	**IC_50_ ± SD^a^ (RF^b^)**		**Treatment**	**IC_50_ ± SD^a^ (RF^b^)**	
	**SW620 (μM)**	**SW620/Ad300 (μM)**		**NCI-H460 (μM)**	**NCI-H460/MX20 (μM)**
Paclitaxel + Gle (1 μM) + Gle (3 μM) + Vera (3 μM)	0.091 ± 0.015 (1.00) 0.067 ± 0.013 (0.74) 0.060 ± 0.020 (0.66) 0.097 ± 0.031 (1.07)	21.190 ± 6.25 (232.86) 1.969 ± 0.160 (21.63)^*^ 0.257 ± 0.072 (2.82)^*^ 0.646 ± 0.173 (7.10)^*^	Topotecan + Gle (1 μM) + Gle (3 μM) + Ko 143 (3 μM)	0.063 ± 0.020 (1.00) 0.060 ± 0.015 (0.95) 0.040 ± 0.021 (0.63) 0.051 ± 0.013 (0.81)	6.010 ± 0.530 (95.49) 6.360 ± 0.127 (100.95) 7.160 ± 1.193 (113.65) 0.520 ± 0.130 (8.25)^*^
Doxorubicin + Gle (1 μM) + Gle (3 μM) + Vera (3 μM)	0.031 ± 0.014 (1.00) 0.033 ± 0.007 (1.06) 0.029 ± 0.012 (0.94) 0.023 ± 0.007 (0.74)	9.950 ± 2.023 (320.97) 2.397 ± 0.041 (77.32)^*^ 0.271 ± 0.020 (8.74)^*^ 0.288 ± 0.155 (9.29)^*^	Cisplatin + Gle (1 μM) + Gle (3 μM) + Ko 143 (3 μM)	1.640 ± 0.185 (1.00) 1.699 ± 0.392 (1.04) 1.513 ± 0.218 (0.92) 1.686 ± 0.152 (1.03)	2.150 ± 0.498 (1.31) 1.926 ± 0.297 (1.17) 2.049 ± 0.187 (1.25) 2.285 ± 0.138 (1.39)
Cisplatin + Gle (1 μM) + Gle (3 μM) + Vera (3 μM)	1.481 ± 0.676 (1.00) 1.266 ± 0.189 (0.85) 1.166 ± 0.079 (0.79) 1.164 ± 0.107 (0.79)	1.514 ± 0.398 (1.02) 1.676 ± 0.138 (1.13) 1.587 ± 0.329 (1.07) 1.851 ± 0.364 (1.25)			

* *P < 0.05 vs. no inhibitor group*.

a *IC_50_ values represented the mean ± SD of three independent experiments performed in triplicate*.

b *Resistance fold (RF) was calculated by dividing the IC_50_ values of substrates in the presence or absence of an inhibitor by the IC_50_ values of parental cells without an inhibitor. Gle, Glesatinib; Vera, verapamil*.

### Glesatinib Did Not Impact the P-gp Expression and Subcellular Localization

The down-regulation or re-localization of P-gp (from cellular membrane to cytosol) may lead to re-sensitization of chemotherapeutics as a result of less extent of efflux or unable to exert its functions (17, 44). We further determined the interaction mechanism of glesatinib with P-gp by examining the P-gp expression and cellular location through Western blotting and immunofluorescence assay. P-gp overexpressing KB-C2 cells were treated with glesatinib at different concentration (0.3, 1, 3 μM for 72 h) or at different time (3 μM for 24, 48, 72 h) and the P-gp expression was examined. SW620/Ad300 cells were treated with 3 μM for 0, 24, 48, 72 h to examine the localization of P-gp. KB-3-1 and SW620 cells were used as negative control in this experiment.

**Figure 2:**
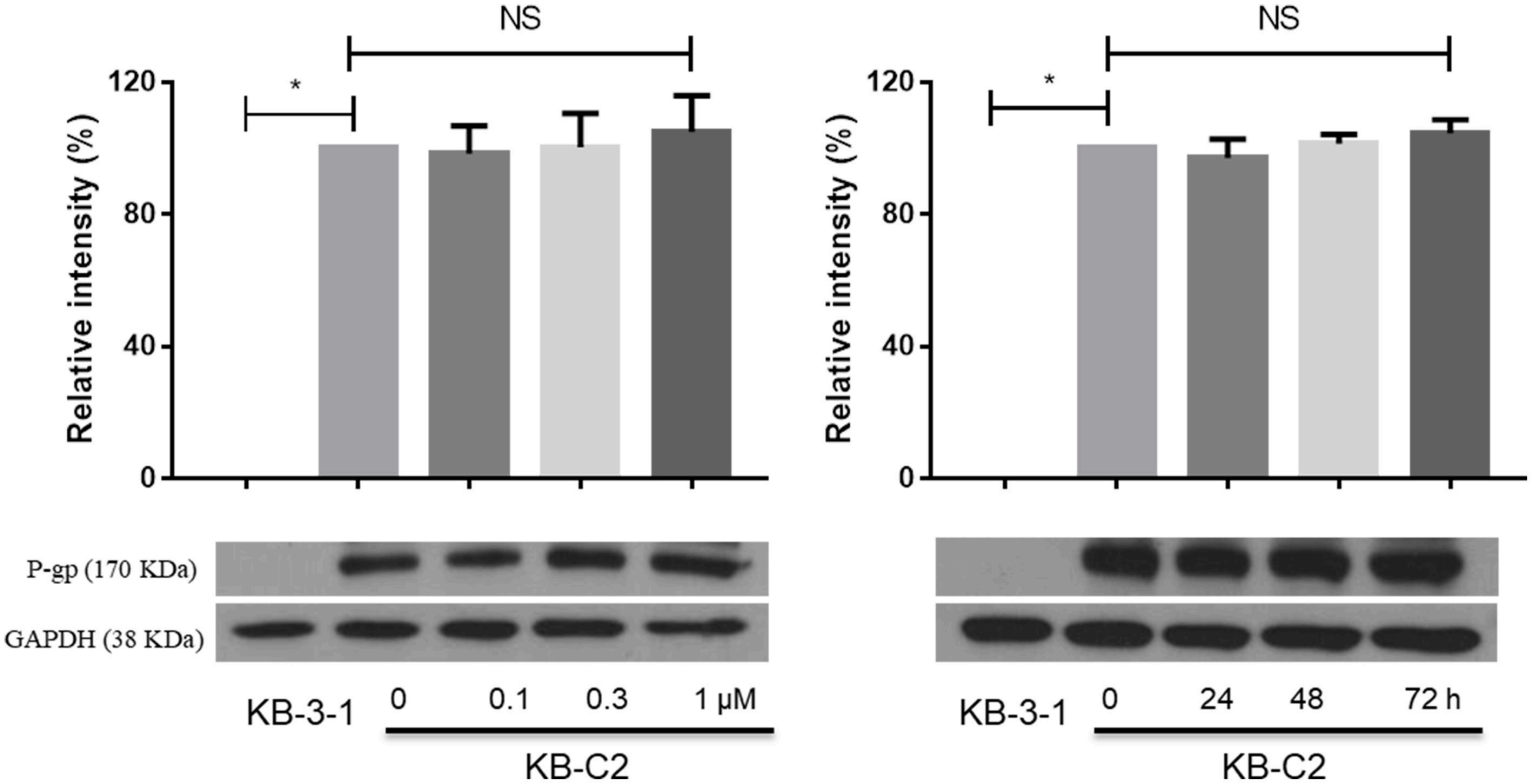
Glesatinib did not affect the protein expression of P-gp transporters in ABCB1 overexpressing cell lines. Detection and relative intensity of ABCB1 expression in KB-C2 cells incubated with 0.3, 1, 3 μM for 72 h and 3 μM for 0, 24, 48, 72 h. Data are mean ± SD, representative of three independent experiments. *p < 0.05, compared with control group.

**Figure 3:**
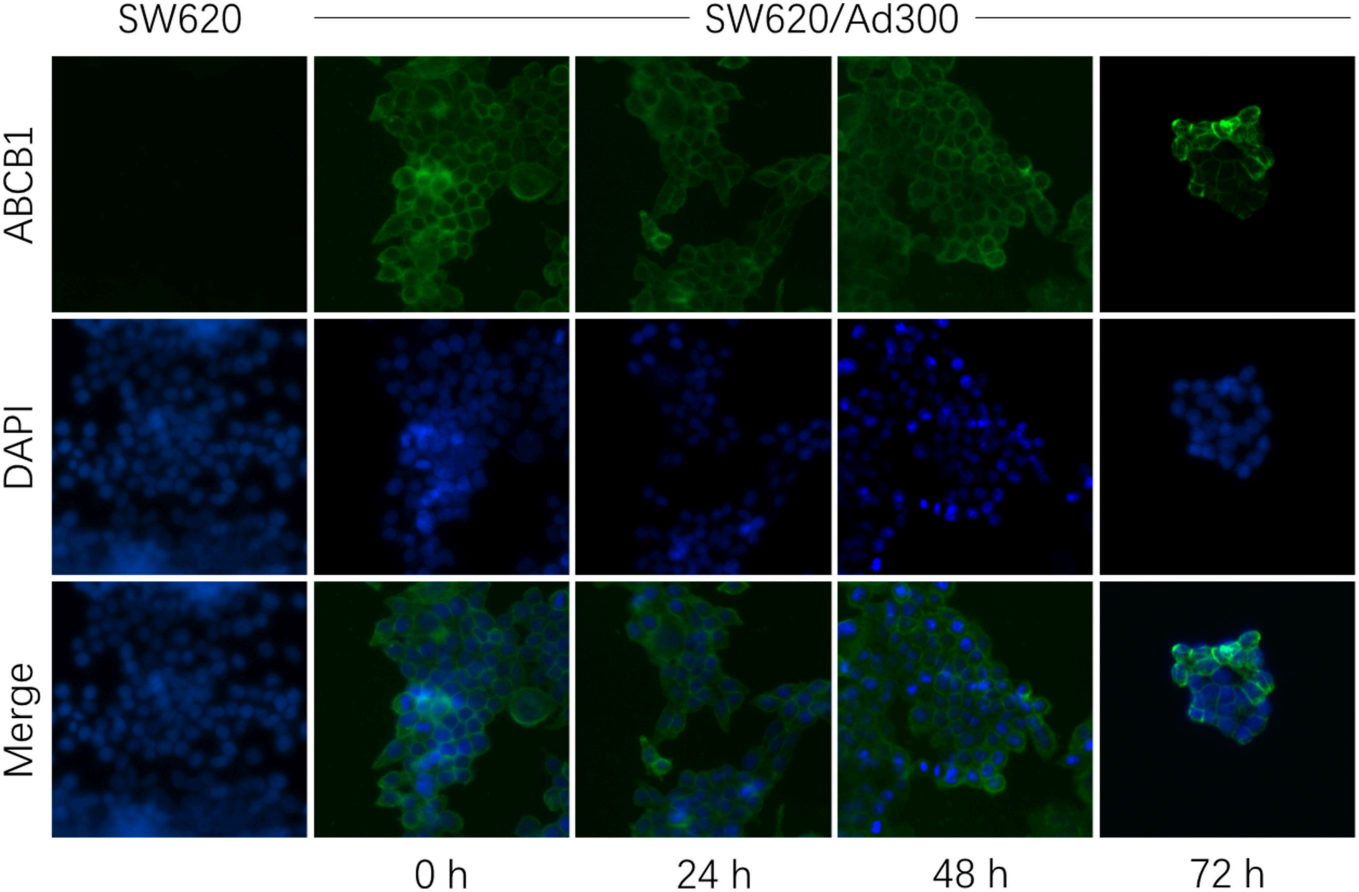
Glesatinib did not affect the localization of ABCB1 transporters in ABCB1 overexpressing cell lines. Sub-cellular localization of ABCB1 expression in SW620/Ad300 cells incubated with 3 μM of glesatinib for 0, 24, 48, and 72 h. ABCB1, green and DAPI (blue) counterstains the nuclei. SW620 cells represented the control group.

As shown in Figure 2, P-gp expression was not impacted by glesatinib either dose- or time-dependently. The immunofluorescence assay results of Figure 3 showed that after treatment of glesatinib, localization of P-gp had not changed and remained to localize on the cell membrane. These results suggested that glesatinib could not impact the expression and localization of P-gp. We next tested the effects of glesatinib to the efflux functions of P-gp.

### Glesatinib Increased the Intracellular [^3^H]-Paclitaxel Accumulation and Inhibited [^3^H]-Paclitaxel Efflux in Cancer Cell Lines Overexpressing P-gp

**Figure 4:**
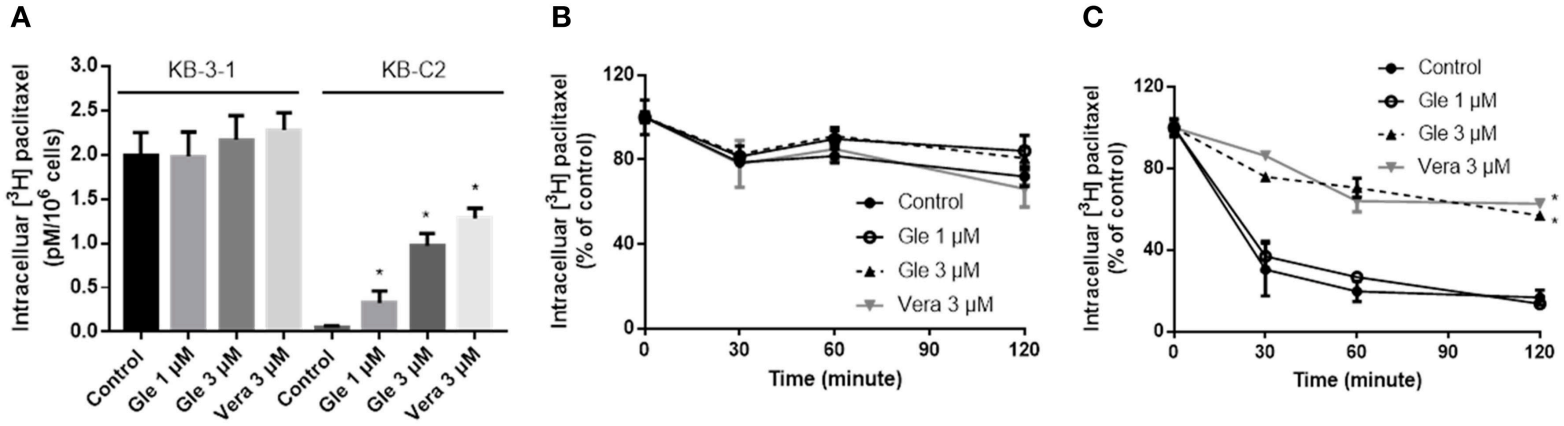
Glesatinib increased the accumulation and inhibited the efflux of [^3^H]-paclitaxel in P-gp overexpressing KB-C2 cells. **(A)** The effect of glesatinib on the accumulation of [^3^H]-paclitaxel in KB-3-1 and KB-C2 cell lines. **(B)** The effect of glesatinib on efflux of [^3^H]-paclitaxel in KB-3-1 and **(C)** KB-C2. Verapamil (3 μM) was used as positive controls. Data are mean ± SD, representative of three independent experiments. *p < 0.05, compared with control group. Gle, Glesatinib; Vera, verapamil.

As glesatinib did not alter either P-gp expression or its localization, we set out to test the transporting function of P-gp by examining the cellular accumulation of radioactive [^3^H]-paclitaxel. As shown in Figures 4A,B, in KB-3-1 cells that barely expressed P-gp, [^3^H]-paclitaxel had not been impacted, and glesatibin had no effects to either the drug accumulation (Figure 4A) or efflux (Figure 4B). While in P-gp overexpressing KB-C-2 cells, [^3^H]-paclitaxel accumulation decreased significantly as shown in Figures 4A,C. Pretreatment of glesatinib may significantly increase the [^3^H]-paclitaxel accumulation and inhibited the drug efflux of P-gp. These results indicated that glesatinib may exert its re-sensitizing effects by thwart the transporting function of P-gp.

### Glesatinib Stimulated the ATPase Activity of P-gp

**Figure 5:**
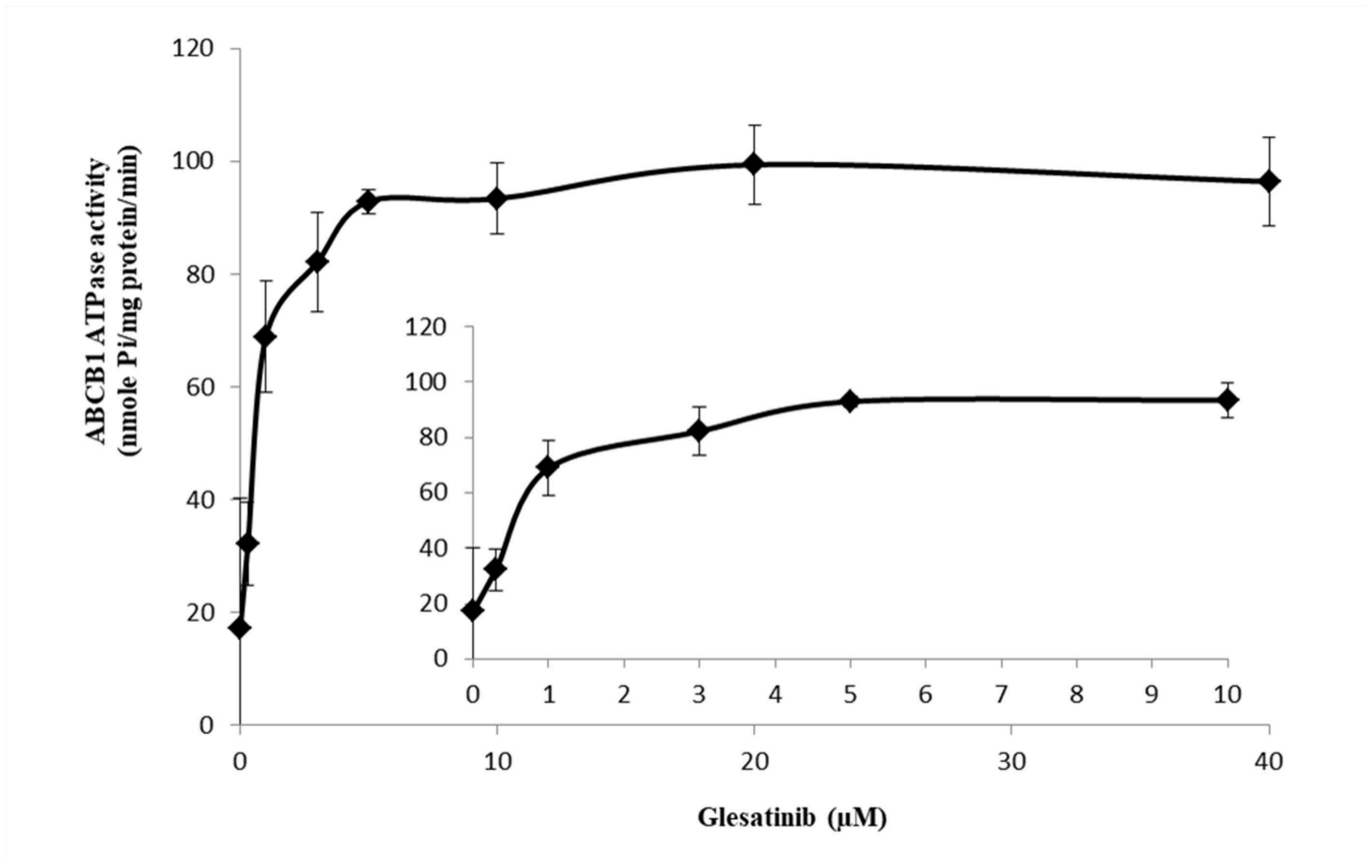
Glesatinib stimulated the ATPase activity of P-gp. Effect of various concentrations of glesatinib on the ATPase activity of P-gp. The inset graphs illustrate the effect of 0–10 μM glesatinib on the ATPase activity of P-gp. Data are mean ± SD, representative of three independent experiments.

ATP hydrolyzed by ATPase was used by P-gp to provide the energy to transport its substrates (45, 46). To further reveal the P-gp inhibitory mechanisms, we determined the effect of glesatinib on the ATPase activity of P-gp transporters by measuring P-gp-mediated ATP hydrolysis in the presence or absence of glesatinib (0–40 μM). As shown in Figure 5, Glesatinib stimulated the ATPase activity of P-gp transporters in a dose-dependent manner. The concentration of 50% stimulation was 3.2 μM, and the maximum stimulation was 5.59-fold greater than that of basal level.

### Induced-Fit Docking (IFD) Simulation Interactions Between P-gp and Glesatinib

**Figure 6:**
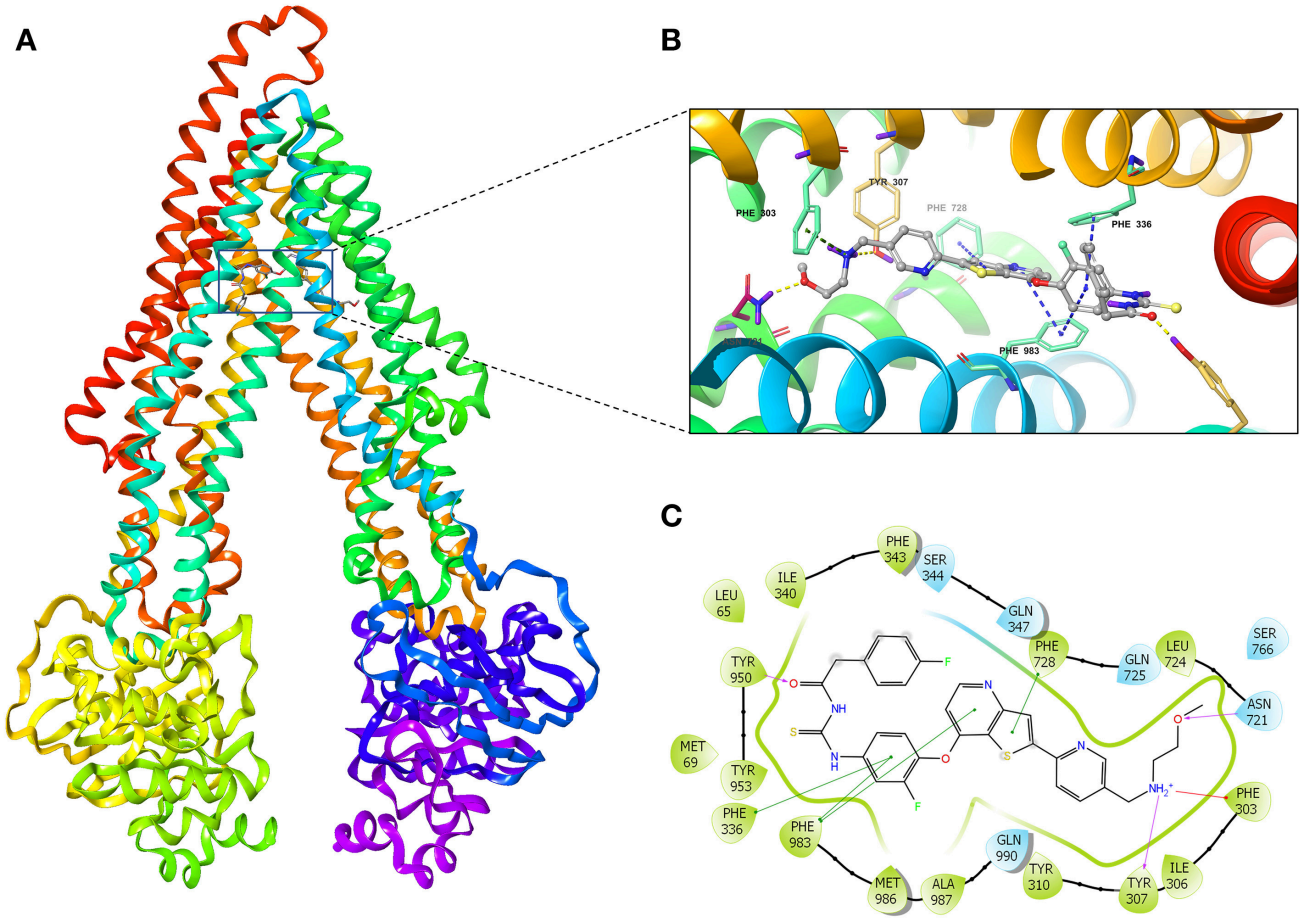
The molecular modeling study of glesatinib with human homology ABCB1. **(A)** Overall view of glesatinib-P-gp complex. **(B)** 3D figure of Docked position of glesatinib within the drug-binding site of human P-gp homology model. Glesatinib was showed as ball and stick mode with the atoms colored: carbon-cyan, nitrogen-blue, oxygen-red, fluorine-green, sulfur-yellow, hydrogen-purple. Important residues were showed as sticks, with the color pattern: carbon-gray, nitrogen-blue, oxygen-red, hydrogen-purple. π-π stacking interactions are indicated with cyan dotted line. π-cation bond is indicated with green dotted line. Hydrogen bonds were showed by the yellow dotted line. **(C)** 2D figure of Docked position of glesatinib within the drug-binding site of human P-gp homology model. The cyan bubbles indicate polar residues and the green bubbles indicate hydrophobic residues. Hydrogen bonds are shown by the purple dotted arrow. π-π stacking interactions are shown by the green lines and π-cation bond is indicated with red line.

We investigated the potential interaction of glesatinib with P-gp by conducting docking analysis. The best docking score of the binding of glesatinib and human P-gp was −12.639 kcal/mol. The best-scored docked position of glesatinib with P-gp was showed in Figure 6. There were two hydrogen bonds between glesatinib and human P-gp, including the hydrogen binding between the amide group of glesatinib and Tyr950 (C = O…HO-Tyr950), in addition with the hydrogen bond between the methoxy group and Asn721 (H_3_C-O…H_2_N-Asn721). The fluorophenyl group of glesatinib has π-π interaction with both Phe336 and Phe983 of P-gp protein. The thienopyridine group has π-π interaction with the residues Phe728 and Phe983. Interestingly, the acidic microenvironment of tumor (47) could result in the ionization of glesatinib, and the amine cation could form a hydrogen bond with Tyr307 and a π-cation bond with Phe303. These formed various bonds between glesatinib and human P-gp may finally lead to the collapsed P-gp.

## Discussion

ABC transporter P-gp functions as the protective enzyme that pumps out xenobiotics including many chemotherapeutics that are its substrates, causing MDR in cancers (3). To counter that, many P-gp inhibitors have been developed and some of them have been tested in clinical trials, while all of them have failed to get approved by US FDA due to severely adverse effects (48, 49). Recent studies indicate that certain TKIs may work as regulators of P-gp (2, 50), either inhibiting its expression (51) or impact its functions (35). Combinations of these TKIs and chemotherapeutics hold promising potential in the treatment of MDR cancers.

In this work, we found that MET/SMO dual inhibitor glesatinib, a drug candidate that is now under clinical trials, antagonized P-gp mediated MDR in cancer cells overexpressing P-gp. As shown in SW620/Ad300 and KB-C2 cells, glesatinib could antagonized P-gp mediated resistance by significantly reducing the IC_50_s of doxorubicin, paclitaxel and colchicine, while had no effects to cisplatin which was not a substrate of P-gp. To confirm these effects were mediated by P-gp, we further tested its reversal effects to P-gp transpected HEK293 cells. Glesatinib exhibited similar effects in HEK293/ABCB1 cells, indicating the effects were mediated by regulating P-gp. We further confirmed that glesatinib did not affect the expression and sub-cellular localization of P-gp, while it could stimulate ATPase, similar as P-gp inhibitor verapamil (45). Importantly, our results showed gleastinib significantly increased the intracellular accumulation of [^3^H]-paclitaxel and suppressed the efflux effects, which may contribute to the increased cytotoxic effects when used by combination. Finally, the docking study indicated that glesatinib might have strong interaction with P-gp via hydrogen bonds and π-π interaction, leading to the efflux inhibition. This docking result may provide valuable information to develop glesatinib derivatives for better targeting and/or binding.

In conclusion, MET/SMO dual inhibitor Glesatinib antagonized P-gp mediated MDR by inhibiting its efflux functions. This work provided important information for further clinical trials.

## Author Contributions

QC and Z-SC: conception and design. QC, C-YC, H-LG, NJ, SS, SA, and Z-SC: development of methodology. QC, C-YC, H-LG, PG, and NJ: acquisition of data. QC, C-YC, H-LG, NJ, and Z-SC: analysis and interpretation of data. QC, C-YC, LR, YY, D-HY, and Z-SC: writing, review, and/or revision of the manuscript. All authors read and approved the final manuscript.

### Conflict of Interest Statement

The authors declare that the research was conducted in the absence of any commercial or financial relationships that could be construed as a potential conflict of interest.
